# Comparison of Cardiac and Vascular Parameters in Powerlifters and Long-Distance Runners: Comparative Cross-Sectional Study

**DOI:** 10.5935/abc.20180167

**Published:** 2018-12

**Authors:** Diego Vidaletti Silva, Gustavo Waclawovsky, Ana Beatriz Kramer, Cinara Stein, Bruna Eibel, Guilherme Brasil Grezzana, Maximiliano Isoppo Schaun, Alexandre Machado Lehnen

**Affiliations:** 1 Instituto de Cardiologia - Fundação Universitária de Cardiologia (IC/FUC), Porto Alegre, RS - Brazil; 2 Faculdade Sogipa de Educação Física, Porto Alegre, RS - Brazil

**Keywords:** Hypertrohy,Ventricular, Exercise, Exercise MovementTechniques, Blood Pressure, Resistance Training, Running/physiology

## Abstract

**Background:**

Cardiac remodeling is a specific response to exercise training and time exposure. We hypothesized that athletes engaging for long periods in high-intensity strength training show heart and/or vascular damage.

**Objective:**

To compare cardiac characteristics (structure and function) and vascular function (flow-mediated dilation [FMD] and peripheral vascular resistance [PVR]) in powerlifters and long-distance runners.

**Methods:**

We evaluated 40 high-performance athletes (powerlifters [PG], n = 16; runners [RG], n = 24) and assessed heart structure and function (echocardiography), systolic and diastolic blood pressure (SBP/DBP), FMD, PVR, maximum force (squat, bench press, and deadlift), and maximal oxygen uptake (spirometry). A Student’s t Test for independent samples and Pearson’s linear correlation were used (p < 0.05).

**Results:**

PG showed higher SBP/DBP (p < 0.001); greater interventricular septum thickness (p < 0.001), posterior wall thickness (p < 0.001) and LV mass (p < 0.001). After adjusting LV mass by body surface area (BSA), no difference was observed. As for diastolic function, LV diastolic volume, wave E, wave e’, and E/e’ ratio were similar for both groups. However, LA volume (p = 0.016) and BSA-adjusted LA volume were lower in PG (p < 0.001). Systolic function (end-systolic volume and ejection fraction), and FMD were similar in both groups. However, higher PVR in PG was observed (p = 0.014). We found a correlation between the main cardiovascular changes and total weight lifted in PG.

**Conclusions:**

Cardiovascular adaptations are dependent on training modality and the borderline structural cardiac changes are not accompanied by impaired function in powerlifters. However, a mild increase in blood pressure seems to be related to PVR rather than endothelial function.

## Introduction

Exercise training induces cardiovascular adaptations secondary to changes in blood pressure as well as other hemodynamic and metabolic changes in response to physical exertion. These adaptive changes can induce left ventricular (LV) hypertrophy in the long run.^1^ Some authors claim that borderline physiological and anatomical changes occur as part of an adaptive process of high-performance training and they have sparked off debate on their implications.^2^ They postulate that volume overload generally increases LV pumping ability producing eccentric hypertrophy while, in contrast, pressure overload decreases ventricular cavity size producing concentric hypertrophy. Moreover, peripheral vascular resistance (PVR) is an important factor of cardiac overload by specifically modulating LV afterload. Furthermore, the endothelium is central to vasodilation by producing nitric oxide (NO), which is a vasodilator and has a direct effect on PVR. Therefore, it is important to highlight that after exercise there is a stimulation of NO production and eNOS phosphorylation, which contributes directly to a reduction in PVR.^3,4^

Aerobic exercise increases shear stress leading to increased release and synthesis of NO and higher active muscle vasodilation.^5^ LV pressure overload is reduced over time.^6^ However, high-intensity resistance training such as weightlifting and powerlifting involves a number of very slow-speed contractions that produce transient mechanical compression of resistance vessels, increasing PVR and LV pressure overload during exercise.^7^ It has been postulated that chronic increase in afterload induces the parallel addition of new sarcomeres in the myocardium leading to concentric ventricular hypertrophy.^8^ Yet, this form of ventricular hypertrophy has not been demonstrated in strength training athletes,^9^ and it is thus an inconsistent finding.

Given the limited body of evidence in support of these cardiovascular adaptations as well as concerning endothelial function and PVR in strength athletes, this study aimed to compare structural and functional cardiac changes in powerlifters and long-distance runners. Secondarily, we compared endothelium-dependent vasodilation and PVR in these athletes. Our hypothesis is that athletes engaging in high-intensity strength training for long periods of time show changes in cardiac structure associated with reduced cardiac function when compared to long-distance runners. Furthermore, long-time exposure to high-intensity strength training could lead to a reduction of endothelial function caused by pressure overload.

## Methods

### Study participant selection and groups

The study convenience sample comprised 40 male individuals aged 18-40 years. We selected athletes of powerlifting (powerlifters group [PG], n = 16) and long-distance (over 10 km) running events (runners group [RG], n = 24). Eligible athletes were those competing for at least 3 years. Individuals with any medical condition in the preceding 6 months; those not competing in the preceding 6 months; those on use of illicit (doping) substances in the last 12 months; or those who refused to sign an informed consent were excluded.

The study sample was recruited using an open invitation at training sites (gyms, health clubs and sports centers) and selected after applying the inclusion criteria. Participants were assessed as follows: on the first visit they underwent blood pressure assessment, echocardiographic assessment, brachial artery flow-mediated dilation, peripheral vascular resistance assessments. In addition, they were administered a comprehensive questionnaire with questions about training including time of training experience; performance timeline; any awards/prizes; current training routine (volume, intensity, and duration of weekly training sessions, frequency of competitive participation, rest times, etc.) among others. On the next day, they underwent a maximum load test; and on the last visit (48 hours later), they underwent a maximum oxygen uptake test. All assessments were carried out within the same period of time (8 a.m. to 11 a.m.).

### Blood pressure assessment

Blood pressure measurements were taken using a semi-automatic blood pressure monitor (OMROM 705CP), with the participant in a seated position with both feet on the floor, after a 10-minute rest; the cuff was placed and adjusted to the arm circumference. In a completely quiet room, blood pressure measurements were taken in duplicate on both arms, and the higher value of these readings was used in the study.

### Echocardiographic examination

Transthoracic echocardiographic examinations were performed by an echocardiography specialist (G.B.G.). An ultrasound device (EnVisor CHD, Philips, Bothell, WA, USA) equipped with a sector transducer probe (2-4 MHz) was used to obtain longitudinal, cross-sectional, two-dimensional 2- and 4-chamber, and M module images. Continuous-wave, pulsed-wave, and color Doppler techniques were used to examine ventricular tissues and walls. All images were stored and sent to a second echocardiography specialist (D.P.K.) for blind evaluation of images. Body surface area (BSA) was calculated using Du Bois method.^10^

### Brachial artery flow-mediated dilation and peripheral vascular resistance

We used a high-resolution two-dimensional Doppler ultrasound device (EnVisor CHD, Philips, Bothell, WA, USA) equipped with a high-frequency (7-12 MHz) linear vascular transducer probe and electrocardiographic imaging and monitoring software. Flow-mediated dilation (FMD) measurements were taken with the participants in the supine position, and a properly fitting pressure cuff was placed on the arm 5 cm above the cubital fossa.^11^ Baseline brachial artery longitudinal diameters were assessed. Following that, the occlusion cuff was inflated to 50 mmHg above the systolic blood pressure (SBP) for 5 minutes and then deflated. Brachial artery diameters were measured for 60 seconds after deflation of the cuff. All analyses were performed offline and brachial artery measurements were made at the end of diastole (at R-wave peak on the electrocardiogram). FMD responses were expressed as percentage change from the baseline brachial artery diameter.

PVR was calculated from mean blood pressure (MBP) and baseline blood flow obtained in the FMD test (PVR = MBP/baseline blood flow in mmHg/cm.s^-^).

### Maximum load test

Maximum strength was assessed in the one-repetition maximum test (1-RM) for the squat, bench press and deadlift exercises, which are specifically performed at competitions, and through the total sum of these three exercises (total load). Distance runners attended a familiarization session within 48 hours of the test when the order of strength exercises and proper performance were introduced. For the 1-RM, the participants performed the maximum number of repetitions with the proposed load, up to a maximum of 10 repetitions. Exercise loads were increased according to Lombardi (1989)up to a point where participants were able to perform only one repetition with a maximum of 3 attempts to achieve the maximum load.

### Maximum oxygen uptake

Maximum oxygen uptake (VO_2_peak or VO_2_max) was assessed through cardiopulmonary exercise test on a treadmill with respiratory gases collected (VO2000 model, Inbramed, Porto Alegre, Brazil). Powerlifters attended a familiarization session within 48 hours of the test where test procedures were introduced (Bruce protocol and mask placement for gas collection). The highest value, either VO_2_ peak or VO_2_ max was recorded at the end of the test as VO_2_ max.

### Statistical analyses

We performed the Shapiro-Wilk test to test normality of the data and homogeneity of variance was tested using Levene's test. All results are described as mean ± SD and confidence interval. We conducted Student's t Test for independent samples to assess differences between groups and calculated Pearson’s linear correlation coefficients (α = 0.05 for all tests). All statistical analyses were performed using SPSS Statistics (version 21 for Windows).

## Results

The participants had similar age and height (Table 1). However, all anthropometric measurements for PG were greater compared to distance RG. In turn, Table 2 shows loads for the squat, bench press, and deadlift exercises and total load (total sum of these three exercises). For all types of exercises, weight loads were higher in PG than RG as expected. The total load was greater by ~133% in PG than RG. The differences remained unchanged when loads were adjusted for body mass.

**Table 1:** General characteristics of the study participants

	PG (n = 16) Mean ± SD (95% CI)	RG (n = 24) Mean ± SD (95% CI)	p-value
Age (years)	29.9 ± 4.4 (27.5–32.2) Min 20 and Max 36	28.7 ± 5.7 (26.3–31.1) Min 18 and Max 40	0.490
Body mass (kg)	99.2 ± 21.5 (87.6–110.7) Min 75 and Max 135	71.7 ± 9.2 (67.7–75.6) Min 58 and Max 84	< 0.001
Height (cm)	176 ± 0.8 (172–181) Min 164 and Max 195	175 ± 0.8 (172–179) Min 161 and Max 193	0.736
Chest circumference (cm)	113.2 ± 13.4 (106–120.4) Min 94.5 and Max 144	86.9 ± 8.6 (83.2–90.5) Min 61 and Max 100	< 0.001
Waist circumference (cm)	95.1 ± 12.9 (88.2–102) Min 78 and Max 117	78.6 ± 5.7 (76.2–81.1) Min 69 and Max 92	< 0.001
Duration of training (years)	5.12 ± 2.0 (4.0–6.2) Min 3 and Max 10	7.8 ± 2.6 (6.7–8.9) Min 3 and Max 10	0.001
Weekly duration of training (days)	3.9 ± 1.0 (3.3–4.4) Min 3 and Max 5	5.4 ± 1.0 (4.9–5.8) Min 3 and Max 7	< 0.001
Daily duration of training (min/day)	69.3 ± 14.4 (61.7–77.0) Min 60 and Max 90	98.7 ± 28.6 (86.6–110.8) Min 60 and Max 120	0.001

PG: powerlifters group; RG: long-distance runners group. Weekly number of training sessions and session average time correspond to the average duration for the last 3 months. Differences between means were assessed using Student’s t Test for independent samples.

**Table 2:** Maximum load test results in absolute values and adjusted for body mass

	PG (n = 16) Mean ± SD (95% CI)		RG (n = 24) Mean ± SD (95% CI)	p-value
Squat (kg)	212.2 ± 46.4 (187.4–236.9) Min 140 and Max 302		98.9 ± 27.1 (87.4–110.6) Min 56 and Max 160	< 0.001
Squat/body mass	2.16 ± 0.27 (2.01–2.30) Min 1.6 and Max 2.6		1.37 ± 0.30 (1.24–1.50) Min 1.0 and Max 2.3	< 0.001
Bench press (kg)	145.5 ± 32.9 (127.9–163.1) Min 110 and Max 220		59.0 ± 16.5 (52.0–66.0) Min 40 and Max 94	< 0.001
Bench press/body mass	1.49 ± 0.26 (1.35–1.62) Min 1.1 and Max 2.1		0.81 ± 0.17 (0.74–0.89) Min 0.6 and Max 1.2	< 0.001
Deadlift (kg)	239.0 ± 66.5 (203.6–274.5) Min 150 and Max 370		102.4 ± 27.8 (90.6–114.2) Min 53 and Max 140	< 0.001
Deadlift/body mass	2.43 ± 0.49 (2.16–2.69) Min 1.5 and Max 3.1		1.45 ± 0.41 (1.28–1.63) Min 0.6 and Max 2.0	< 0.001
Total load (kg)	596.8 ± 137.4 (532.6–670.1) Min 413 and Max 890		260.4 ± 43.8 (241.9–278.9) Min 191 and Max 341	< 0.001
Total load/body mass	6.07 ± 0.89 (5.59–6.55) Min 4.4 and Max 7.4		3.64 ± 0.48 (3.44–3.85) Min 2.6 and Max 4.6	< 0.001

PG: powerlifters group; RG: long-distance runners group. Differences between means were assessed by Student's t Test for independent samples.

Table 3 shows hemodynamic and cardiopulmonary parameters. Powerlifters had higher resting SBP (~10%) and resting DBP (~12%); the absolute differences between the two groups were 13.6 mmHg and 10.1 mmHg, respectively. Resting heart rate was higher in PG compared to RG (~19%, Δ15.7 bpm). VO_2_max was much higher in RG than PG (~65%): the highest VO_2_ max value among powerlifters was lower than the lowest VO_2_ max value among runners.

**Table 3:** Hemodynamic and cardiopulmonary parameters

	PG (n = 16) Mean ± SD (95% CI)	RG (n = 24) Mean ± SD (95% CI)	p-value
Resting SBP (mmHg)	130.0 ± 8.2 (124.5–134.0) Min 120 and Max 140	116.4 ± 8.6 (112.8–120.1) Min 110 and Max 140	< 0.001
Resting DBP (mmHg)	82.1 ± 6.9 (78.1–68.1) Min 70 and Max 95	72.0 ± 6.5 (69.3–74.8) Min 60 and Max 80	< 0.001
Resting heart rate (bpm)	80.4 ± 7.5 (76.0–84.8) Min 69 and Max 94	64.7 ± 10.3 (60.3–69.1) Min 45 and Max 90	< 0.001
Maximum heart rate (bpm)	180.2 ± 13.7^‡^ (173.2–188.2) Min 158 and Max 209	184.3 ± 14.7‡ (178.1–190.5) Min 167 and Max 224	0.403
VO_2_ max (mL.kg^-1^.min^-1^)	33.9 ± 7.5 (29.6–38.9) Min 24 and Max 43	56.0 ± 7.3 (52.7–62.1) Min 45 and Max 74	< 0.001
VCO_2_ max (mL.kg^-1^.min^-1^)	36.6 ± 9.3 (31.2–42.0) Min 24 and Max 57	58.0 ± 7.5 (55.2–61.6) Min 45 and Max 87	0.028
Pulmonary ventilation (L.min^-1^)	103.5 ± 17.6 (93.3–113.7) Min 76 and Max 136	112.4 ± 14.9 (106.1–118.7) Min 85 and Max 157	0.106

SBP: systolic blood pressure; DBP: diastolic blood pressure; PG: powerlifters group; RG: long-distance runners group. VO_2_: oxygen uptake; VCO_2_: carbon dioxide production. Differences between means were assessed by Student’s t Test for independent samples.

‡ p < 0.05 vs. baseline value within the same group.

Table 4 shows the echocardiographic results. As for cardiovascular adaptations, aorta diameter, left atrium (LA) diameter, right ventricle diameter, LV systolic diameter, and LV diastolic diameter were similar in both groups. However, PG showed greater interventricular septum thickness (Δ2.4 mm) and posterior wall thickness (Δ1.2 mm). They also showed greater LV mass (Δ46.5 g), but this difference disappeared after adjusting for BSA. As for diastolic function, LV diastolic volume, transmitral E wave, e' wave, and E/e' ratio were similar in both groups. However, LA volume (~22%), and LA volume adjusted for BSA (~40%) were found in PG, when compared to RG, but they were all within normal ranges. Although PG showed some degree of anatomical remodeling and different diastolic function parameters compared to RG, systolic function reflected in LV systolic volume, ejection fraction, and ejection fraction calculated by Simpson’s rule were similar in both groups. Of the 40 participants, 9 (22.5%) had physiological ventricular hypertrophy in response to exercise; 10 (all powerlifters) had interventricular septum thickness greater than 11 mm. Of the 27 participants with LV mass greater than 225 g and LV mass adjusted by BSA greater than 115g/m^2^, 13 (82%) were PG and 14 (63%) RG.

**Table 4:** Echocardiographic parameters

	PG (n = 16) Mean ± SD (95% CI)		RG (n = 24) Mean ± SD (95% CI)	p-value
**Anatomical structures**				
Aorta diameter (mm)	31.3 ± 3 (29.7–32.9) Min 25 and Max 36		32.0 ± 2.7 (30.8–33.2) Min 29 and Max 38	0.410
LA diameter (mm)	36.0 ± 2.5 (34.6–37.3) Min 30 and Max 39		35.6 ± 2 (34.7–36.5) Min 32 and Max 39	0.632
RV diameter (mm)	20.3 ± 1.2 (19.6–20.9) Min 18 and Max 22		20.5 ± 2 (19.6–21.4) Min 16 and Max 25	0.689
LV end-systolic diameter (mm)	30.7 ± 3.9 (28.6–32.8) Min 23 and Max 37		30.2 ± 2.9 (28.9–31.5) Min 25 and Max 36	0.671
LV end-diastolic diameter (mm)	53.4 ± 3.3 (51.5–55.3) Min 45 and Max 60		53.7 ± 3.3 (52.2–55.2) Min 45 and Max 57	0.770
Interventricular septum thickness (mm)	12.0 ± 1.0 (10.6–12.3) Min 10 and Max 14		9.6 ± 0.4 (9.4–9.9) Min 9 and Max 10	< 0.001
Ventricular posterior wall thickness (mm)	10.4 ± 0.9 (9.9–10.9) Min 9 and Max 12		9.1 ± 0.5 (8.9–9.4) Min 8 and Max 10	< 0.001
LV mass (g)	282.2 ± 73.4 (243–321.4) Min 150 and Max 406		235.7 ± 26.0 (224.2–247.3) Min 179 and Max 276	< 0.001
LV mass/BSA (g/m^2^)	135.6 ± 24.9 (136.1–133.6) Min 90 and Max 173		127.8 ± 16.9 (120.3–135.4) Min 104 and Max 166	0.262
**Diastolic function**				
End-diastolic volume (mL)	145.0 ± 18.9 (134.9–155.1) Min 92 and Max 173		138.1 ± 17.2 (130.5–145.8) Min 92 and Max 160	0.251
Transmitral E-wave velocity	0.83 ± 0.15 (0.75–0.90) Min 0.6 and Max 1.1		0.91 ± 0.15 (0.84–0.97) Min 0.6 and Max 1.3	0.124
e’ wave	0.15 ± 0.03 (0.13–0.17) Min 0.1 and Max 0.2		0.17 ± 0.34 (0.15–0.19) Min 0.1 and Max 0.2	0.062
E/e’ ratio	5.69 ± 1.05 (5.12–6.24) Min 4.1 and Max 8.0		5.56 ± 1.76 (4.78–6.34) Min 3.0 and Max 11.8	0.808
Transmitral A-wave velocity	0.35 ± 0.03 (0.33–0.37) Min 0.3 and Max 0.4		0.38 ± 0.04 (0.36–0.40) Min 0.3 and Max 0.5	0.047
LA volume (mL)	35.7 ± 8.5 (31.2–40.2) Min 22 and Max 53		43.6 ± 10.2 (39.1–48.2) Min 32 and Max 76	0.016
LA volume/BSA (mL/m^2^)	16.7 ± 4.1 (14.5–18.8) Min 11 and Max 27		23.4 ± 4.6 (21.4–25.5) Min 16 and Max 37	< 0.001
**Systolic function**				
End-systolic volume (mL)	38.0 ± 11.2 (31.9–44) Min 18 and Max 58		34.8 ± 9.3 (30.6–38.9) Min 22 and Max 54	0.348
Ejection fraction (%)	73.0 ± 4.5 (70.5–75.4) Min 67 and Max 80		74.3 ± 4.6 (72.3–76.3) Min 65 and Max 86	0.383
Ejection fraction by Simpson’s rule (%)	71.6 ± 4.8 (69.1–74.2) Min 62 and Max 79		72.7 ± 5.9 (70.1–75.4) Min 61 and Max 81	0.568

PG: powerlifters group; RG: long-distance runners group; LA: left atrium; RV: right ventricle; LV: left ventricle; BSA: body surface area. Differences between means were assessed using Student’s t Test for independent samples.

**Figure 1:**
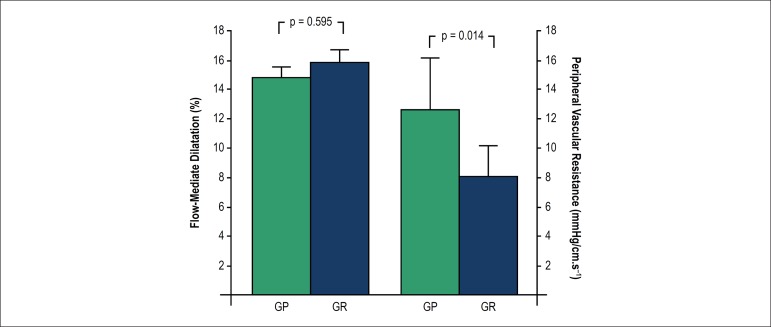
Flow-mediated dilation measurements and peripheral vascular resistance. PG: powerlifters group, RG: long-distance runners group. The differences were assessed by Student’s t Test for independent samples.

Figure 1 shows FMD (%) and PVR measurements. Interestingly, FMD values were similar in both groups ([PG] 14.7 ± 2.3 vs. [RG] 15.9 ± 2.5%). However, PG had higher PVR values compared to RG ([PG] 12.6 ± 5.3 vs. [RG] 8.2 ± 3.8 mmHg/cm.s^(-1)^, Δ35%).

The correlations between training parameters and echocardiographic and cardiopulmonary variables in PG are displayed in Table 5. There was a direct correlation between interventricular septum thickness and weight load in the deadlift, squat, and total load. Interestingly, no correlation was found with time of exposure, i.e., duration in years of strength training among powerlifters. SBP levels were directly correlated with training intensity; and DBP showed a stronger correlation with duration of strength training. For runners, interventricular septum thickness and resting heart rate were inversely correlated with VO_2_max and duration of strength training (Table 6).

**Table 5:** Pearson linear correlation coefficients between training parameters and echocardiographic /cardiopulmonary variables (PG = 16)

	Total load (kg)	Duration of strength training (years)	Weekly duration of training (days)	Daily duration of training (min/day)
Interventricular septum thickness (mm)	0.733^†^	0.411	0.286	0.212
Posterior ventricular wall thickness (mm)	0.680^†^	0.365	0.274	0.225
LV mass (g)	0.689^†^	0.407	0.213	0.248
Resting heart rate (bpm)	0.706^†^	0.505	–0.149	0.201
Baseline SBP (mmHg)	0.029	0.377	0.258	0.453
Baseline DBP (mmHg)	0.490	0.762^†^	0.581^*^	0.151
VO_2_ max (mL.kg^-1^.min^-1^)	–0.459	–0.093	0.048	0.135
VCO_2_ max (mL.kg^-1^.min^-1^)	–0.623^*^	–0.133	–0.051	–0.022

PG: powerlifters group; 1-RM: one-repetition maximum test; LV: left ventricle, SBP: systolic blood pressure; DBP: diastolic blood pressure; VO_2_: oxygen uptake; VCO_2_: carbon dioxide production. Significance level

† p < 0.001 and

* p < 0.05.

**Table 6:** Pearson linear correlation coefficients between training parameters and echocardiographic variables (RG = 24)

	VO_2_ max (mL.kg^-1^.min^-1^)	VCO_2_ max (mL.kg^-1^.min^-1^)	Pulmonary ventilation (L.min^-1^)	Duration of strength training (years)	Weekly duration of training (days)	Daily duration of training (min/day)
Interventricular septum thickness (mm)	–0.640^*^	0.362	0.303	–0.630^*^	0.150	0.136
Posterior ventricular wall thickness (mm)	0.001	–0.016	0.209	0.260	–0.139	0.032
LV mass (g)	–0.140	–0.137	–0.015	–0.110	–0.248	–0.100
Resting heart rate (bpm)	–0.650^*^	–0.550	–0.414	–0.659^*^	–0.163	–0.244
Baseline SBP (mmHg)	0.177	0.311	0.341	–0.074	–0.023	–0.212
Baseline DBP (mmHg)	0.183	0.279	0.258	0.701	0.254	–0.101

RG: long-distance runners group; LV: left ventricle; SBP: systolic blood pressure; DBP: diastolic blood pressure; VO_2_: oxygen uptake, VCO_2_: carbon dioxide production. Significance level

* p < 0.05.

Finally, FMD measurements were directly proportional to training intensity (% 1-RM) in PG and weight load for the squat (Table 7). For RG, no correlation of FMD values was found with cardiopulmonary variables and resting heart rate. Furthermore, FMD values were correlated with duration of powerlifting training (years) and daily duration of training session. However, this same correlation was not seen among runners.^12^

**Table 7:** Pearson linear correlation coefficients between training parameters and brachial artery flow-mediated dilation measurements

	Squat (kg)		Bench press (kg)		Deadlift (kg)		VO_2_ max (mL. kg^-1^.min^-1^)		Resting heart rate (bpm)		Duration of strength training (years)		Weekly duration of training (days)		Daily duration of training (min/ day)	
	PG	RG	PG	RG	PG	RG	PG	RG	PG	RG	PG	RG	PG	RG	PG	RG
FMD (%)	0.710^†^	0.351	0.242	0.165	0.654^†^	–0.383	0.073	–0.349	0.489	–0.107	0.688^*^	0.165	0.491	–0.123	0.770^†^	–0.079

PG: powerlifters group; RG: long-distance runners group; FMD: flow-mediated dilation. Significance level

† < 0.001,

* p < 0.05.

## Discussion

Our study found that, compared with long-distance runners, powerlifters showed greater interventricular septum thickness, LV posterior wall thickness and LV mass. However, after adjusting for BSA, no difference was observed in LV mass.Cardiac function was similar in powerlifters and runners. Together, these parameters suggest that specific cardiac remodeling may occur as a result of training, but with no impairment of cardiac functions. A major finding of our study was similar FMD measurements in both powerlifters and runners despite PVR being higher in powerlifters.^12^ Although our findings are comparative and derive from a cross-sectional design, they suggest that high-intensity strength training does not necessarily cause damaging cardiovascular changes as it has been generally believed.

### Cardiac parameters

Regarding cardiac parameters (anatomical structure, and diastolic and systolic function), the echocardiographic assessments showed increased interventricular septum thickness with slight or no chamber diameter reduction and slight increase in posterior wall thickness in powerlifters compared to runners. These changes may be because powerlifting involves a great amount of slow-speed contractions using high loads close to the maximum^13^ in daily training sessions leading to LV pressure overload.

As for the cutoff values, several studies with high-performance athletes have used to determine pathological hypertrophy cutoff values of 12-13 mm for maximum interventricular septum thickness and 55-60 mm for end-diastolic dimension, as described below.Whyte (2004) examined 306British elite male athletes (judo, n = 22; skiing, n = 10; pole vault, n = 10; kayak, n = 11; rowing, n = 17; cycling, n = 11; power lifters, n = 29; triathlon, n = 51; modern pentathlon, n = 22; middle distance, n = 45; rugby, n=30; tennis, n = 33; swimming, n = 19) and found interventricular septum thickness > 13 mm in ~3.0% of them. Riding (2012) examined 836 athletes (soccer, n = 586; basketball, n = 75; volleyball, n = 41 and handball, n = 35) and found interventricular septum thickness > 12 mm and typical features of concentric left ventricular hypertrophy in ~2.0%. Pelliccia (1999) examined 1,309 Italian elite athletes engaged in different sporting disciplines (soccer, n = 119; gymnastics, n = 87; rowing, n = 80; tennis, n = 64;basketball, n = 62; track and field, n = 59; alpine skiing, n = 59; shooting, n = 57; handball, n = 56; cycling, n = 49; water polo, n=43; ice hockey, n = 42; cross-country skiing, n = 41; canoeing, n = 39; rugby, n = 39; skating, n = 36; fencing, n= 35; yachting, n = 33; swimming, n = 29; equestrian sports, n = 24; karate, n = 24; volleyball, n = 21; bobsledding, n = 17; boxing, n = 15; wrestling, n = 14; judo, n = 13; luge, n = 13; field hockey, n = 13; table tennis, n=11; pentathlon, n = 7; weight-lifting, n = 7; golfing, n = 6; baseball, n=5; triathlon, n = 3; motor-racing, n = 3; body-building, n=3; other modalities n = 72) and found interventricular septum thickness > 13 mm in 1.1% of them. Moreover, they also found that 45% and 14% of the athletes studied exhibited end-diastolic dimension > 55 mm and > 60 mm, respectively. Thus, if we use these cutoffs, despite some anatomical cardiac changes, none of the study participants showed cardiac dimensions consistent with pathological hypertrophy. However, it is important to note a strong correlation between weight loads lifted in the squat and total load and cardiac dimensions including septum thickness, posterior wall thickness, and LV mass. Yet again, a possible explanation is that powerlifting involves a great amount of slow-speed contractions using high loads close to the maximum leading to a pressure overload.^9-17^

With regard to LV mass, Gardin et al.,^18^ reported values of 225 g and 115 g/m² adjusted by BSA in individuals chronically exposed to pressure overload. LV mass was also measured in our study and we found values of 282 g and 135 g/m^2,^ among powerlifters. Interestingly, runners also showed high LV mass (236 g and 128 g/m^(2 )^adjusted by BSA). Regardless of the training modality, cardiac remodeling occurred in response to exercise training in both groups. Though still controversial, echocardiographic measurements indexed to BSA allow to comparing individuals of different body sizes. BSA is affected by fat mass, and fat mass is neither correlated with nor predicts LV mass.^19^ An alternative approach is to adjust echocardiographic parameters for lean mass. However, accurate measurements are not widely available and substitute methods such as skin-fold thickness measurements are relatively inaccurate.^20,21^

Diastolic function assessment in the study revealed consistently normal values in long-distance runners.^22^ In contrast, lower LA volume and transmitral A-wave velocity measures were found in powerlifters although these values were within normal limits. The difference of LA volume measures between both groups was ~22%, and it was even more pronounced after adjustment for BSA (~40%). D’Andrea et al.,^23^ and coworkers have assessed LA volume and BSA-indexed LA volume in 350 endurance athletes and 245 strength athletes.^23^ For BSA-indexed measures, these authors defined values between 29 and 33 mL/m^2^ as mild LA enlargement and values greater than 33 mL/m^2^ as moderate LA enlargement. Thus, our results were all below the cutoff values set in D'Andrea et al.,^23^ As for LV systolic function assessed through estimates of ejection fraction and ejection fraction calculated by Simpson's rule, the echocardiographic assessment showed values within the normal range in all cases.

### Blood pressure

The association of aerobic training with lower resting blood pressure is well established.^24,25^ But a growing body of evidence shows that strength training can have a similar effect on blood pressure,^26^ though there is not yet a consensus in the literature.^27^ However, high-intensity strength training has been reported to negatively affect blood pressure. A meta-analysis showed that training modalities that basically consist of strength training (powerlifting*,* bodybuilding, and Olympic weightlifting) are associated with a higher risk of high blood pressure with mean SBP of 131.3 ± 5.3 mmHg and mean DBP of 77.3 ± 1.4 mmHg.^28^ These values are consistent with those found in our study (SBP 130.0 ± 8.2 and DBP 82.1 ± 6.9 mmHg).

### Vascular function

FMD measurements were similar in both powerlifters and runners. This is an interesting finding given that these two training modalities have different biomechanical and metabolic characteristics. Exercise training has been shown as an effective means for the improvement of endothelium-dependent vasodilation capacity.^29^ Among high-performance athletes, long-distance runners with above average normal cardiac function show lower arterial stiffness, lower oxidative stress, and increased endothelium-dependent dilation^30^ capacity when compared to sedentary individuals of the same age.^31^ These data suggest that outstanding cardiac performance in athletes may be associated with improved vascular function induced by aerobic exercise training.

It is well known that aerobic exercise improves endothelial function by producing increased shear stress on the vessel walls during exercise.^32^ Yet, it has been suggested that strength training can increase hemodynamic stress due to the mechanical compression of blood vessels during active movements together with excessive vascular tension produced during strength exercises.^7^ Thus, we can speculate that high-intensity strength training could acutely affect endothelium-dependent vasodilation and lead to permanent damage in the long run. In this regard, impaired vascular function has been demonstrated in strength athletes, though it appears to be related to the use of anabolic agents rather than an effect of training.^33,34^

Heffernan et al. found increased forearm reactive hyperemia in healthy young individuals after 6-month strength training.^35^ The most likely explanation for increased endothelium-dependent dilation in strength training is the assumption of the mechanical compression of resistance vessel walls during exercise, followed by blood flow release after cessation of exercise, producing a sharp increase in vessel wall shear stress.^36^ Although training modalities involve different stimuli (running training: increased continuous blood flow; strength training: intermittent compression of the muscles and restoring blood flow) they ultimately produce the same effects on vessel wall shear stress.

It is important to note that, despite increased blood pressure levels and greater posterior wall thickness and LV mass found in our study among powerlifters, they showed no cardiac and endothelial function impairment when compared to runners and all the parameters were above average. Therefore, high blood pressure found in powerlifters seems to be related to increased PVR rather than endothelial function impairment.

### Study strengths and limitations

The key strengths of our study are the use of a homogeneous sample (within each group) and that all echocardiographic images were assessed by two independent examiners, one of them blinded. However, our data should be interpreted with caution due to some limitations including the small sample size (due to recruitment challenges as anabolic steroid use is common among powerlifters and few met our inclusion criteria), and the challenge of recruiting a sample of untrained healthy subjects; however, all parameters evaluated were compared with those findings of other studies and/or current guidelines.

## Conclusion

Our study showed that cardiac remodeling seems dependent on training modalities and not on structural difference, as in BSA-indexed LV mass in both powerlifters and long-distance runners. Systolic and diastolic functions were preserved in both modalities. Powerlifters showed higher resting blood pressure, which can be explained by increased PVR. However, FMD measurements were similar in both groups studied and were well above average. Although our findings are comparative in nature and derive from a cross-sectional design, it is possible to speculate that high-intensity strength training for a significant number of years (~5 years or more) may be associated to borderline structural cardiac changes, though they are not accompanied by reduced cardiac function.
